# Single-cell sequencing of non-infectious and viral uveitis defines distinct mechanisms in ocular inflammation

**DOI:** 10.3389/fimmu.2026.1916535

**Published:** 2026-10-08

**Authors:** Andrei Belean, Shiori Kuramoto, Isabel Risch, Lynn M. Hassman, Atsunobu Takeda, Koh-Hei Sonoda, Nobuyo Yawata, Nicholas C. Borcherding, Michael A. Paley

**Affiliations:** 1 Department of Medicine, Washington University School of Medicine, St. Louis, MO, United States; 2 Department of Ophthalmology, Graduate School of Medical Sciences, Kyushu University, Fukuoka, Japan; 3 Department of Ophthalmology, University of Colorado Anschutz, Denver, CO, United States; 4 Department of Ophthalmology, Faculty of Medicine, Oita University, Yufu, Oita, Japan; 5 Graduate School of Medical Sciences, Ocular Pathology and Imaging Science, Kyushu University, Fukuoka, Japan; 6 Department of Pathology and Immunology, Washington University School of Medicine, St. Louis, MO, United States; 7 Department of Ophthalmology and Visual Sciences, Washington University School of Medicine, St. Louis, MO, United States

**Keywords:** B cells, ocular inflammation and immunology, single cell RNA sequencing, T cells, uveitis

## Abstract

**Introduction:**

Intraocular inflammation can arise from various conditions and can lead to severe vision loss and blindness. The differences in the intraocular immune response between infectious and autoimmune etiologies have not been defined.

**Methods:**

Here we use single-cell RNA sequencing to compare the innate and adaptive immune response across 73 ocular and blood samples from 35 individuals with viral uveitis, autoimmune uveitis, or non-ocular inflammatory disease.

**Results:**

This atlas suggests that viral uveitis is dominated by an influx of myeloid cells that activate cytotoxic CD8 T cells via antigen presentation, while in autoimmune uveitis, dendritic cells appear to activate both type 17 and regulatory CD4 T cells. Paired VDJ sequencing revealed that, while T-cell differentiation is modulated by etiology, B cells initiate similar responses between autoimmune and viral uveitis but differ by the degree of clonal expansion and somatic hypermutation.

**Discussion:**

Collectively, these data identify the differences in innate and adaptive immune responses based on the intraocular stimulus.

## Introduction

Uveitis is an inflammatory disease of the eye that can lead to severe vision loss or blindness if not controlled. This inflammation can occur in any structure of the uvea (the iris, the ciliary body, or the choroid) and can arise from infectious etiologies or from autoimmune processes, including systemic rheumatologic conditions or ocular-specific diseases. Despite the diverse array of etiologies, the measurable ocular manifestation of active inflammation is quite limited, such as anterior chamber flare or vitreous haze that arise from the local production of inflammatory proteins, anterior chamber or vitreous cells that reflect exit of immune cells from ocular tissues into the clear, fluid-filled chambers of the eye, vascular sheathing that is an accumulation of immune cells along blood vessels, and leakage of fluorescein on an ocular angiogram that reflects increased vascular permeability. While patterns of ocular damage can help a clinician focus on a specific disease process, such as heterochromia (iris color loss) and iris atrophy for Fuchs’ uveitis syndrome, these clinical measurements are not able to define the molecular mechanisms that drive inflammatory diseases of the eye.

Since the development and commercialization of single-cell RNA sequencing technologies, several studies have been performed on ocular immune cells to better understand the mechanisms of non-infectious uveitis (1–5). These studies have highlighted the dramatic clonal expansion and formation of tissue-resident populations of T or B cells within the eye, the importance of macrophages in the recruitment of other ocular immune cells, and the multitude of immunologic endotypes. However, the only reference samples in these studies to serve as controls are concurrent blood samples or blood samples from healthy individuals, as the healthy eye does not contain immune cells in the anterior chamber (5). As a result, a comparison between infectious and non-infectious uveitis by single-cell sequencing has not been performed previously.

Herein we compare the ocular immune response of viral uveitis and non-infectious uveitis (NIU), also referred to as autoimmune uveitis. We identify the differences in cellular composition and gene expression signatures between anti-viral and autoimmune responses within the same organ. Moreover, we find that differences in disease etiology leads to divergence of the transcriptional programs of intraocular myeloid cells and T cells but not in B cells. Collectively, this atlas provides additional understanding of the different inflammatory mechanisms that can occur within the human eye.

## Methods

### Participants and sample collection

A total of 73 samples from 35 subjects were analyzed, including two subjects (UV027 and UV215) sampled longitudinally. Clinical metadata are available in **Supplementary Table S1**.

### Viral uveitis cohort

A total of 13 individuals diagnosed with viral uveitis were enrolled. The etiologies included acute retinal necrosis from either varicella zoster virus (*n* = 9), herpes simplex virus 1 (*n* = 1), or herpes simplex virus 2 (*n* = 1), chronic retinal necrosis from cytomegalovirus (*n* = 1), and uveitis from human T-lymphotropic virus-1 (*n* = 1). The participants were recruited between 2020 and 2023 from Kyushu University Hospital in Japan. Viral uveitis was diagnosed based on ocular findings and polymerase chain reaction (PCR) analysis of intraocular fluid samples. The study adhered to the Declaration of Helsinki and received approval from the Institutional Review Board of Kyushu University Hospital (no. 21159). Written informed consent was obtained from all participants. Ocular viral uveitis samples were collected during a clinically indicated vitrectomy. The vitreous perfusion fluid collected during vitrectomy was centrifuged, and the cells were harvested. Paired blood samples were collected via phlebotomy, and peripheral blood mononuclear cells (PBMC) were collected by Ficoll gradient separation. The vitreous cells and PBMCs were resuspended in Cell Banker (Zenogen Pharma, Japan) and cryopreserved in liquid nitrogen.

### Non-infectious uveitis cohort

A total of 21 human participants with non-infectious uveitis were enrolled in accordance with the Declaration of Helsinki principles and using protocols approved by the institutional review board of Washington University in St. Louis (IRB 201704141 and 201912043). Written informed consent was received from the participants prior to their inclusion in the study. For ocular samples, participants with active anterior uveitis, defined by at least 1+ anterior chamber (AC) cell (>6 cells per high-powered field) (6), were enrolled for paired ocular and blood sampling. AC and blood sampling was performed as previously described (5). Briefly, approximately 100–200 μL AC fluid was removed and centrifuged at 400*g* for 5 min, after which the aqueous fluid was removed. Blood samples were obtained by venipuncture, collected into EDTA tubes, and purified by Ficoll–Hypaque density gradient centrifugation. The AC cells and PBMCs were cryopreserved in FBS with 10% DMSO and stored at –140 °C.

### Healthy controls

Blood-only healthy controls and phakic-lens controls were free of ocular inflammatory disease from any etiology.

### Library preparation and sequencing

Frozen AC and vitreous cells were thawed and washed once with FBS and once with PBS with 0.1% BSA. Due to the low AC cell numbers (5,000–30,000 cells), minimal processing was performed to reduce cell loss. Vitreous cells were filtered through a 35-um mesh filter. PBMCs were thawed and washed twice with 10% RPMI and then once with PBS with 0.1% BSA. Viability was >95% by trypan blue exclusion. Single-cell 5′ gene expression cDNA libraries were generated per the 10x Genomics Chromium Single-Cell 5′ Library and Gel Bead Kit v1 and the 10x Chromium Controller (10x Genomics) platform for microdroplet-based, single-cell barcoding by the Genome Technology Access Center at the McDonnell Genome Institute (GTAC@MGI, Washington University in St. Louis). T-cell and B-cell enrichment libraries were generated per the Chromium Single-Cell V(D)J Enrichment Kits for human T cell and human B cell (10X Genomics) using the same input samples. All libraries were sequenced at GTAC@MGI on the NovaSeq Sequencing System (Illumina) with 50,000 target reads per cell for GEX and 5,000 target reads per cell for TCR and BCR enrichment libraries.

### Alignment and preprocessing

Raw sequencing reads were processed with Cell Ranger v8.0.1 multi-command against the GRCh38 human reference for gene expression and the matched human immune receptor references. BCR contigs were further re-aligned and annotated with IgBlast v1.22.0 (7) using the germline reference to produce AIRR-formatted tables. Downstream processing was performed in R v4.5.1 and utilized Seurat v5.3.1 (8). Filtered feature barcode matrices were loaded per capture, with low-quality cells identified as cells with <100 features, >10% mitochondrial content, and the number of read counts > mean plus 2.5 times the standard deviation. Doublets were identified per run using scDblFinder v1.22.0, and predicted doublets were removed prior to integration (9). Per-run reference-based annotations were generated using Azimuth v0.5.0 with the pbmcref on the SCTransform-normalized data and SingleR v2.10.0 (10) with the Human Cell Primary Atlas (11) and the Monaco immune data (12).

### Adaptive immune receptor VDJ annotation

Filtered TCR contigs were combined across runs using scRepertoire v2.5.7 using the combineTCR function with selection of the top-expressing TCRα/β chains per cell. BCR analysis was performed using the Immcantation suite (13, 14). Cells with a single productive heavy-chain sequence with isotype calls were retained. Using shazam v1.3.0 (14), hamming distances between junctions were computed per subject with the clonal distance threshold using a Gaussian mixture model or using density valleys between the two largest modes. Clones were assigned per subject using the scoper v1.3.0 (15) heirachialClones function with the previously calculated threshold. Germline sequences were reconstructed with alakazam v1.4.1 (16), and light chains were then used to resolve subclones. Somatic hypermutation was quantified using the observedMutations function in shazam with counts and frequencies split by the complementarity-determining region (CDR) and framework region (FWR) across both the heavy and light chains. TCR and BCR records were merged into a single per-cell table, with automated removal of the TCR clonal information if the cell was present in both modalities.

### Single-cell processing and integration

Pre-run objects were merged, and a secondary run-aware doublet call was performed to remove any remaining doublets. Stable variable features were calculated across all samples. From these features, V(D)J, ribosomal, mitochondrial, and heat-shock protein genes were removed, and the remaining top 2,000 features were log-normalized and used downstream. Batch correction was performed using the fastMNN function in batchelor v1.24.0 (17) with sequencing run as the batch variable; the number of components and the mutual-nearest-neighbor parameter k are reported in **Supplementary Table S2**. From the fastMNN embedding, a shared-nearest-neighbor graph was built with bluster v1.18.0 using Jaccard edge weights, cosine distance, and Annoy for kNN approximation. Leiden clustering and UMAP were tuned over the grids specified in **Supplementary Table S2**, with three repeats per setting at independent seeds; for each setting, we computed (i) stability (mean pairwise adjusted Rand index across repeats), (ii) graph modularity, (iii) mean silhouette width on the fastMNN embedding, and (iv) the singleton-cluster fraction and selected the highest-scoring configuration as the final partition. The same scoring was used to select UMAP neighbors and distances by maximizing the silhouette of the Leiden labels in UMAP space. Cross-lineage mixing of clusters was corrected using a *post-hoc* per cell score with the Seurat AddModuleScore function using the following markers: T cells—*CD3D/E/G*, *TRAC*, and *TRBC1/2*; NK cells—*KLRF1*, *SH2D1B*, *NCR3*, and *SPON2*; B cells—*CD19*, *MS4A1*, *CD79A/B*, *BANK1*, *MZB1*, and *JCHAIN*; and myeloid cells—*CD14*, *LYZ*, *S100A8/9*, and *FCGR3*. Clusters with minority population fractions exceeding 15% were flagged as mixed. Minority-lineage cells within the mixed clusters were re-assigned to the nearest same-lineage cluster centroid in the fastMNN embeddings using Euclidean distances. The cells were then annotated by a weighted consensus over five evidence sources: (i) reference-based labels (Azimuth, SingleR with HPCA and Monaco references; weight 1 each), (ii) overlap of per-cluster differentially expressed genes with canonical lineage panels (weight 2), (iii) cluster-averaged expression of lineage-discriminating markers (weight 3), (iv) V(D)J chain-type detection rate (TRAV/TRBV vs. IGHV/IGLV; weight +5), and (v) a penalty (weight −3) was applied to T-lineage scores in clusters with V(D)J detection rates below 5%. Reference-specific labels were harmonized to a common vocabulary before voting. NK cells were refined in two stages: a cluster-level rescue when *NKG7/GNLY/KLRD1* signals exceeded myeloid signals at low CD3 and a per-cell recovery within T-cell clusters restricted to cells lacking a detected TCR contig, with the majority subclusters either promoted in place or split as a new cluster ID.

Subset analyses were performed on intraocular cells only and re-integrated using the same pipeline, replacing the full-atlas labels with eye-specific Leiden and consensus annotations. Lens-fiber contaminants were removed before integration by thresholding the summed log-normalized expression of a curated panel of genes, including crystallin and *MIP*/*BFSP*/*LIM2*. Each compartment was built from the eye sub-atlas by selecting cells with the corresponding harmonized broad label (B cell, T cell, and monocyte/macrophage + DC). Before splitting, a rank-based UCell (14) lens-fiber filter (per-batch threshold) was applied at eye level to remove residual crystallin-high cells missed by the bulk threshold. For the myeloid compartment, cells whose clonal amino acid sequence matched the top expanded T/B clones were dropped as carryover/doublet signatures. Surviving cells were re-integrated with fastMNN using a per-sample SCE list, dropping per-sample subsets with fewer than 30 cells; the same *VDJ/MT/RPL/RPS/HSP* filtering was applied. Leiden and UMAP were re-tuned per compartment using the same SNN + score framework, with compartment-specific grids (B cell, T cell, and myeloid) tuned to each compartment’s biology and reported in **Supplementary Table S2**. A *post-hoc* cluster-level contamination filter then scored each Leiden cluster against an expected canonical panel and one or more off-target panels (T/B/myeloid/stromal); clusters whose top off-target score exceeded the expected score (and reached ≥0.20 detection) were removed, and the compartment was re-clustered on the embedding restricted to surviving cells.

### Differential analysis of genes and cells

Three primary contrasts were pre-specified: eye versus blood, autoimmune versus viral, and granulomatous versus non-granulomatous. For each contrast, DGE was run globally and per cluster using two complementary strategies. Pseudobulk DESeq2-utilized raw counts were aggregated within each (sample × group) combination using AggregateExpression (8, 18). Per-cluster pseudobulk matrices with at least two samples per group and at least four total samples were retained. Genes with fewer than 10 counts in at least two samples were filtered. DESeq2 was run with design ~ group, and results were extracted by named contrast; per-cluster and global tables were concatenated. For Wilcoxon rank-sum, FindMarkers with logfc.threshold = 0.25, min.pct = 0.1, requiring at least three cells in each group within a cluster. Robustness was assessed by removing each autoimmune subdiagnosis and repeating the analysis. Agreement with the full differential gene analysis was quantified using the Jaccard index of significant genes, Spearman correlation of log2-fold change, and retention of the 50 most significant genes. Single-cell gene-set scoring for the MSigDB Hallmark and curated (C2) gene sets was performed in escape v2.6.1 (19) using the UCell algorithm (20). Differential pathway activity for eye versus blood and autoimmune versus viral contrasts was tested with Wilcoxon rank-sum and with Benjamini–Hochberg-adjusted *p*-values reported. In parallel, for each pseudobulk DGE contrast, significant genes (FDR < 0.05) were used for over-representation analysis using clusterProfiler v4.16.0 (21). Neighborhood-level differential abundance was tested with miloR v2.4.1 (22). A Milo object was built on the fastMNN embedding, and two contrasts were evaluated: (i) eye versus blood across the full dataset and (ii) NIU versus viral, restricted to eye-derived cells. Sample identity for countCells was a combination of the sample and time point. calcNhoodDistance was computed on the PCA-stored fastMNN embedding, and testNhoods returned per-neighborhood logFC and spatial-FDR. Neighborhoods were annotated with Leiden clusters and broad cell types using annotateNhoods.

### Per-cluster pseudobulk principal component analysis

For each compartment, subject-level pseudobulk matrices were built per (subject × cluster) with the Seurat function AggregateExpression, requiring at least 20 cells per (subject, cluster) for myeloid/B cells and 30 for T cells. Genes with fewer than 10 counts across the pseudobulk were filtered. For the B-cell compartment, only plasma cells (cluster 0) and memory B cells (cluster 1) met the minimum subject coverage in the autoimmune-versus-viral contrast. DESeq2 variance-stabilizing transformation was applied with vst controlling for phenotype (23), the top 2,000 highly variable genes were selected by row variance, and principal components were computed on the scaled VST matrix using prcomp in the stats v4.5.0 R package. The first five components were retained, and PC1 was sign-flipped so that the viral centroid sat at positive PC1 (PC1_oriented). The autoimmune-versus-viral PC1 split per cluster was tested by using Welch *t*-test on PC1_oriented against the phenotype.

### Cross-compartment PC1 bridge and clone-group sharpening

Subject-level myeloid PC1 was bridged to subject-level T-cell PC1. Per-cluster PC1-oriented scores were aggregated first to subject and cluster as a cell-count-weighted mean across subject and timepoint and then to subject as a cell-count-weighted mean across clusters. Marginal correlations were computed pooled and per-etiologies with bootstrap 95% confidence intervals over 2,000 iterations and permutation null *p*-values over 1,000 iterations. A rank-based partial Spearman estimate was calculated alongside the Pearson correlation. Partial correlations controlled for phenotype by residualization. Robustness was assessed by removing each patient, in turn, and repeating the analysis. Ligand–receptor coupling between myeloid and T-cell clusters was inferred per etiology using the LIANA (v0.1.14) R package with the rank-aggregated consensus across method–method scores (24).

### TCR specificity and motif discovery

T-cell receptor specificity was assigned at the cell level using two orthogonal sources. Known antigen specificity was inferred by matching CDR3-beta against the VDJdb (25) release 2026-05-16, restricted to *Homo sapiens*, with the self-antigen filter applied. Matches were called by exact CDR3-beta plus allele-stripped V-gene identity. The curated pathogen panel was VZV, CMV, HTLV-1, HSV-1, HSV-2, EBV, influenza A, and SARS-CoV-2. The canonical HLA-B*27-associated pathogenic TCR was flagged by *TRAV21* paired with a CDR3-beta matching the [YF]S[TS] flanking motif (26). Unbiased motif discovery used GLIPH2 (27) via the new R-implemented immGLIPH (v0.99.5) R package on intraocular TRB sequences with the human_v2.0_CD48 reference, minimum CDR3 length 8, minimum group size 3, 1,000 simulations, and 200 bootstrap iterations for the NIU-versus-Viral enrichment test with Benjamini–Hochberg correction. Per-clone TCRβ generation probabilities were computed with OLGA (28) via the new immLynx (v1.0.0) R package wrapper, using the prebuilt humanTRB IGoR-derived V(D)J recombination model (29). Calculations were restricted to in-frame, non-stop-codon CDR3β sequences from intraocular T cells. We scored every GLIPH motif cluster from intraocular TRB sequences (≥2 unique CDR3β per cluster) across 12 features in five axes. Disease bias used a per-cluster NIU-vs.-viral Fisher exact test (BH-corrected), the precomputed NIU-enrichment FDR, and the NIU cell fraction. Clonal convergence and expansion used distinct contributing subjects, the count of CDR3β present in ≥3 cells, and total cluster cells. Antigen selection used the median and minimum log_10_OLGA Pgen (humanTRB model, CDR3β-only). Phenotype context used the modal Leiden T-cell cluster of cluster members, its MiloR differential-abundance logFC (positive = NIU-up), and a row-mean Z-score over an autoimmune receptor panel (*CTLA4*, *CXCR3*, *IL2RA*, *CD44*, *ITGA4*, *CD69*, and *PTPRC*). Cross-compartment engagement used the count of top-quartile NIU-biased LIANA myeloid-to-T-cell pairs targeting that dominant cluster, plus mean network eigen-centrality from the GLIPH graph. Each feature was oriented, so higher values favored novelty, ranked across clusters with average ties, and min–max scaled to [0, 1]. Missing values collapsed to a neutral 0.5. The composite was a weighted mean (Fisher FDR 2.5, n_cells 2.0, NIU enrichment 1.5, NIU fraction 1.5, subject breadth 1.2, dominant-cluster DA logFC 1.0, receptor Z 1.0, log-Pgen 0.6, LR engagement 0.5, and network centrality 0.3), and clusters were ranked ascending by negative score. A comprehensive set of motifs and TCRs is available in **Supplementary Table S3**.

### BCR lineage architecture and cross-cluster sharing

B-cell lineages were taken from the per-subject scoper assignments and classified as expanded if they contained at least three unique cells. For each expanded lineage, member cells were mapped to their B-cell Leiden cluster using the compartment-specific reintegration, and lineages with members spanning at least two of the five B-cell clusters were flagged as multi-cluster. Disease association with multi-cluster lineage membership was tested by logistic regression of cell-level multi-cluster status on disease phenotype, with log_10_(clone size) as a covariate. The size-adjusted odds ratio for phenotype was reported as the primary effect estimate. Maximum-parsimony trees were built with dowser v2.4.0 (30) on phangorn v2.12.1 (31) backbones and rendered with ggtree v3.16.0 (32), with tips shaped by heavy-chain isotype (IgA and IgG) from the AIRR-formatted table and colored by Leiden B cluster assignment. Cluster-to-cluster sharing was visualized as chord diagrams with circlize v0.4.16 (33), using a symmetric matrix of shared cells per ordered pair of B clusters across all expanded lineages within each disease group.

The dominant isotype per clone was assigned as the majority c_call_heavy value among IGH cells in the clone, collapsed from IGHM, IGHD, IGHG, and IGHA to IgM, IgD, IgG, or IgA, with ties broken by alphabetical order. Per-clone mean somatic hypermutation was computed as the per-cell sum of region-stratified mutation frequencies (mu_freq_cdr_r_heavy, mu_freq_cdr_s_heavy, mu_freq_fwr_r_heavy, and mu_freq_fwr_s_heavy) from using observedMutations in the shazam R package, averaged across cells in the clone. Clones spanning at least two of the five B-cell Leiden clusters were labeled shared and the remainder non-shared. Per-disease isotype distributions across shared and non-shared clones were tested using *χ*^2^ with Monte Carlo *p*-values (*B* = 10,000), and cross-disease homogeneity was assessed by the Cochran–Mantel–Haenszel test. Per-clone mean somatic hypermutation (SHM) was compared between shared and non-shared clones within each disease using the Wilcoxon rank-sum test, with Cliff’s *δ* from effectsize v1.0.1 as the effect size. The cohort-level SHM contrast between viral and autoimmune samples was tested using the Wilcoxon test on per-subject SHM means to avoid clone-level pseudo-replication.

### Statistical analysis

Unless otherwise stated, group comparisons used the Wilcoxon rank-sum test; neighborhood-level DA used the quasi-likelihood framework in miloR with spatial-FDR correction. Benjamini–Hochberg FDR was used for multiple comparison correction across gene-level (DESeq2/Wilcoxon), pathway-level, and differential abundance tests. The significance thresholds were as follows: *α* = 0.05 for gene-level tests, spatial-FDR <0.1 for miloR neighborhoods, and *α* = 0.05 for gene set analysis.

## Results

### Cohort and integrated single-cell atlas

To resolve the immune compartments engaged at the site of ocular inflammation, we generated paired 5′ single-cell RNA-sequencing with matched TCR and BCR libraries on aqueous humor (non-infectious/autoimmune uveitis samples) or vitreous humor (viral uveitis samples) and peripheral blood mononuclear cells (PBMCs) from healthy controls and patients (**Figure 1A**). The cohort included 73 samples across three groups: 46 autoimmune/idiopathic (23 eye and 23 PBMC), 20 viral (13 eye and seven PBMC), and seven healthy controls (PBMC only). Viral etiologies included varicella zoster virus (VZV), herpes simplex virus (HSV), cytomegalovirus (CMV), and human T-lymphotropic virus-1. A full breakdown of the cohort is available in **Supplementary Table S1**. Per-sample QC and doublet removal across 71 sequencing runs yielded 432,360 high-quality cells. The atlas resolved 11 immune populations spanning T, NK, B, plasma, myeloid, dendritic, platelet, and erythroid lineages (**Figure 1B**). Canonical lineage markers tracked cleanly with the cluster identities (**Figure 1C**).

**Figure 1:**
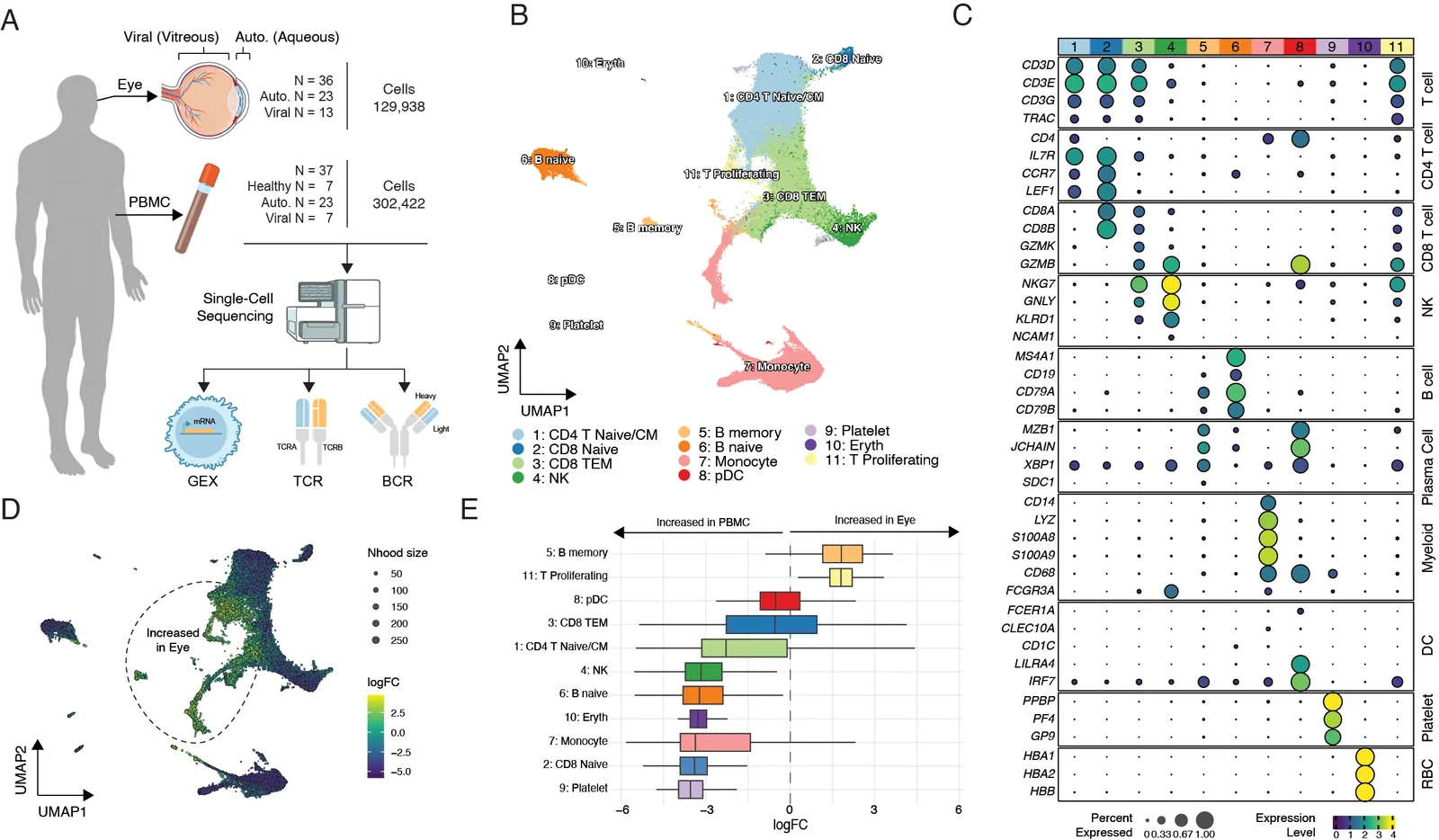
Integrated single-cell atlas of paired intraocular fluids and peripheral blood immune cells across uveitis etiologies. **(A)** Paired eye and peripheral blood mononuclear cells (PBMCs) were collected from subjects across healthy control, viral, and autoimmune/idiopathic uveitis etiologies and processed for 5′ single-cell RNA sequencing with paired VDJ (TCR + BCR) libraries. **(B)** UMAP embedding of all post-QC cells (*n* = 432,360 cells across 71 runs) colored by final cell-type annotation. **(C)** Dot plot of canonical lineage markers across the 11 annotated clusters. Dot size encodes the fraction of cells expressing each gene; color encodes scaled mean expression. **(D)** Differential abundance analysis between eye and PBMC using a subject-paired design. UMAP of neighborhoods colored by log_2_ fold-change (eye vs. PBMC); dot size encodes neighborhood size. **(E)** Per-cell-type distribution of neighborhood log2-fold changes from **(D)**. Neighborhoods are grouped by majority cell-type annotation and ordered by median logFC; positive values indicate enrichment in eye, and negative values indicate enrichment in PBMC.

To localize compartment-specific shifts in composition, we performed differential abundance testing between the eye and PBMC under a subject-paired design (**Figure 1D**). The eye compartment preferentially harbored B memory cells, proliferating T cells, and plasmacytoid dendritic cells. PBMC samples carried higher fractions of naive CD8 T cells, naive B cells, NK cells, monocytes, erythrocytes, and platelets (**Figure 1E**). The intraocular compartment thus carried an antigen-experienced phenotype, while peripheral blood retained a naive and innate cell bias.

### Autoimmune and viral uveitis drive distinct transcriptional signatures

Higher-resolution sub-clustering of the integrated atlas resolved 13 transcriptional states across the intraocular cells (**Figure 2A**). This sub-analysis included 85,697 and 32,594 cells from viral and autoimmune/idiopathic uveitis, respectively. Canonical markers confirmed the lineage assignments, with *CD3D*, *CD8A*, *CD4*, and *FOXP3* marking the T-cell compartment, *MS4A1* and *MZB1* marking B and plasma cells, *CD68* and *FCGR1A* marking the myeloid compartment, *NKG7* and *KLRD1* marking NK cells, and *LILRA4* and *IL3RA* marking plasmacytoid dendritic cells (pDCs) (**Figure 2B**). UMAP overlays of canonical cell type markers are available in **Supplementary Figure S1**. Cluster-defining genes cleanly separated the 13 populations, with each cluster carrying a distinct top-marker signature (**Figure 2C**).

**Figure 2:**
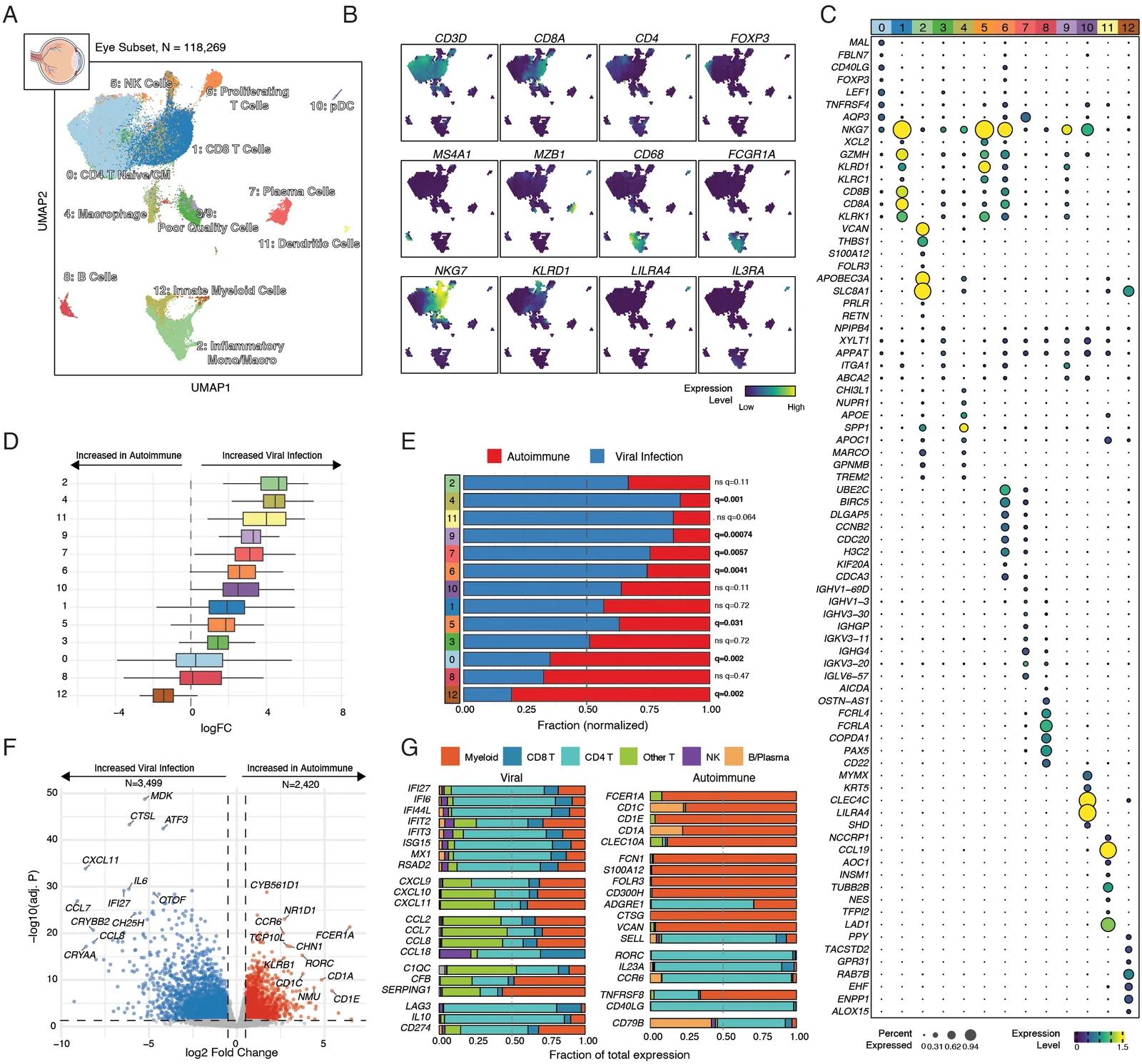
Autoimmune vs. viral immune signatures across paired intraocular fluid. **(A)** UMAP embedding of all post-QC cells colored by Leiden cluster with consensus cell-type annotations overlaid. **(B)** Feature plots of canonical lineage markers projected on the UMAP from **(A)**. Markers cover T-cell (*CD3D*, *CD8A*, *CD4*, and *FOXP3*), B/plasma cell (*MZB1* and *MS4A1*), myeloid (*CD68* and *FCGR1A*), NK (*NKG7* and *KLRD1*), and pDC (*LILRA4* and *IL3RA*. **(C)** Dot plot of top cluster-defining genes filtered at adjusted *p <*0.05 and pct_diff >0.4. Dot size encodes the fraction of cells expressing each gene. Color encodes scaled mean expression. **(D)** Per-cluster distributions of pseudobulk log_2_ fold changes for the autoimmune vs. viral contrast. Boxes shifted to the left indicate genes increased in autoimmune samples. Boxes shifted to the right indicate genes increased in viral samples. **(E)** Normalized stacked bars of per-cluster autoimmune (red) and viral (blue) cell fractions after correcting for unequal sampling depth. **(F)** Volcano plot of the global pseudobulk autoimmune vs. viral contrast. Points with adjusted *p <*0.05 and absolute log_2_ fold change >1 are colored blue (viral) or red (autoimmune). Top genes by adjusted *p*-value are labeled. **(G)** Lineage contribution to the leading viral- and autoimmune-enriched genes from **(F)**. Each horizontal bar is one gene, partitioned into the fraction of its total expression contributed by each broad lineage (color), summed across the clusters in **(A)**.

To localize the disease axis to specific cell populations, we ran pseudobulk differential expression within each cluster comparing autoimmune and viral uveitis. The per-cluster log_2_ fold-change distributions showed that monocyte/macrophage and activated macrophage clusters carried the largest and most consistent shifts between groups, while naive and central-memory T cells were more transcriptionally stable across the contrast (**Figure 2D**). The cellular composition itself differed across groups: viral eye cells were dominated by myeloid and cytotoxic effector populations, while autoimmune samples contributed proportionally more cells with naive and memory T-cell phenotypes (**Figure 2E**).

The global pseudobulk contrast identified 9,391 differentially expressed genes at adjusted *p <*0.05, with 3,499 transcripts increased in viral and 2,420 increased in autoimmune samples at greater than one log_2_ fold change (**Figure 2F**; **Supplementary Table S4**). Viral-enriched transcripts converged on a coordinated antiviral effector program. Top hits included type I and II interferon-stimulated genes (*IFI27*, *IFIT2*, *IFIT3*, and *RSAD2*), CXCR3-ligand chemokines (*CXCL9*, *CXCL10*, and *CXCL11*), monocyte-recruiting chemokines (*CCL7*, *CCL8*, and *CCL18*), classical complement components (*C1QC*, *CFB*, and *SERPING1*), and inhibitory immune checkpoints (*LAG3*, *IL10*, and *CD274*). Decomposing each leading gene’s signal across lineages showed that this program was distributed rather than restricted to a single compartment (**Figure 2G**). The myeloid compartment made the dominant contribution to the complement components and a substantial share of the chemokines, the interferon-stimulated genes were expressed across compartments and were contributed mainly by the abundant CD4 T-cell pool, and unconventional T cells added a notable fraction of the CXCR3-ligand chemokines. This pattern fits a myeloid-led chemokine and complement response layered on a tissue-wide interferon signature at the site of ocular inflammation.

Autoimmune-enriched transcripts split into coherent programs with distinct cellular sources (**Figure 2G**). A CD1-restricted antigen-presentation program (*CD1A*, *CD1C*, *CD1E*, *CLEC10A*, and *FCER1A*, with *CD1B* and *CD207* in the wider hit list) was contributed almost entirely by the myeloid lineage, consistent with the expansion of cDC2- and Langerhans-like antigen-presenting cells. A classical and inflammatory monocyte program (*FCN1*, *S100A12*, *FOLR3*, and *VCAN*) was likewise myeloid-derived. In contrast, a type 17 axis, marked by *RORC* and accompanied by *IL23A* and *CCR6* (**Figure 2G**) and by *IL17F*, *IL26*, *IL22RA2*, *IL23R*, KLRB1 (*CD161*), *GPR15*, and *CSF2* across the wider hit list, was contributed predominantly by CD4 T cells. Variable IGH, IGK, IGL, TRA, and TRB gene segments dominated the long tail of autoimmune-enriched hits and likely reflect oligoclonal lymphocyte expansion within the inflamed eye (**Supplementary Table S4**). Excluding each NIU sub-diagnosis, in turn, did not change the direction or membership of the intraocular signature (**Supplementary Figure S2**, Spearman rho 0.99 across informative folds, no sign reversals). The etiology axis is, therefore, not an artifact of pooling clinically distinct diseases. This allowed us to ask where within the intraocular compartment the difference originates, starting with myeloid cells.

### The intraocular myeloid compartment partitions by disease etiology and predicts divergent T-cell instruction

We next isolated and analyzed the intraocular myeloid cells from patients with autoimmune uveitis and viral uveitis. Unsupervised clustering recovered nine subsets that cover the myeloid lineage (**Figure 3A**). The monocyte and macrophage compartments included classical inflammatory monocytes (cluster 0), intermediate monocytes (cluster 6), *FOLR2*+ tissue-resident macrophages (TRM, cluster 1), *TREM2*+ lipid-associated macrophages (LAM, cluster 2), and a residual macrophage population (cluster 3). The dendritic compartment contained plasmacytoid DCs (pDC, cluster 4), Langerhans-like/conventional type 2 dendritic cells (cDC2) (cluster 5), cDC1 (cluster 7), and mature regulatory DCs (mregDC and cluster 8). Canonical markers confirmed each identity (**Figure 3B**). Pathway module scoring revealed that each subset runs a distinct intrinsic program (**Figure 3C**; **Supplementary Table S5**). *TREM2*+ LAM scored highest for cholesterol/NOTCH and autophagy/hypoxia modules, in line with their lipid-handling phenotype. Classical inflammatory monocytes dominated the cell cycle/NFKB module. cDC1 and mregDC showed the strongest p53/complement and apoptosis/hypoxia activity. These programs separated the lineage at the level of cell identity rather than at the level of disease context.

**Figure 3:**
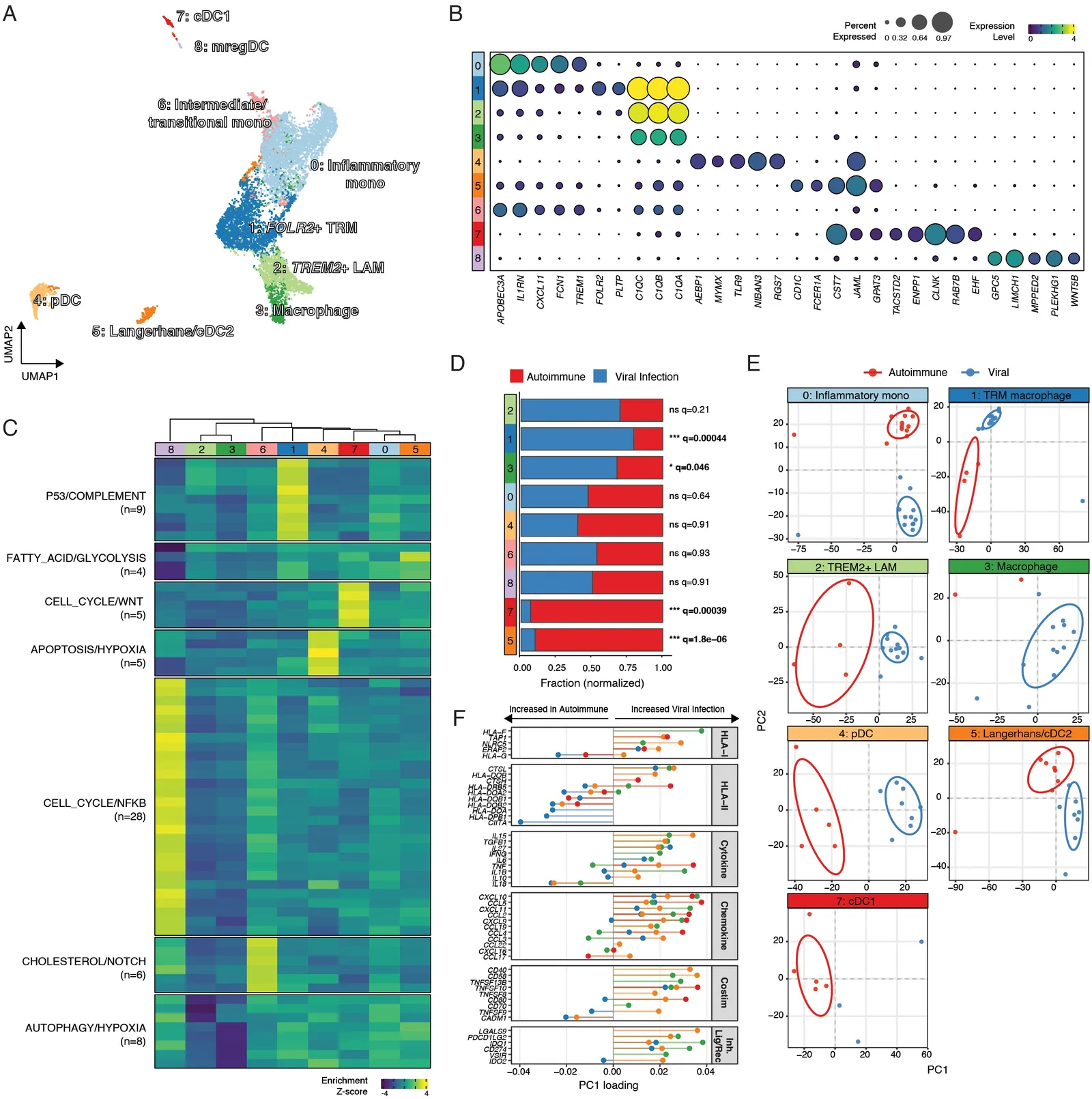
Intraocular myeloid cells diverge by disease etiology in autoimmune versus viral uveitis. **(A)** UMAP embedding of intraocular myeloid cells aggregated across autoimmune (*n* = 19) and viral (*n* = 7) uveitis samples (12,288 cells passing QC). **(B)** Dot plot of top differential markers per cluster. Dot size encodes the fraction of cells expressing each gene, and color encodes scaled mean expression. **(C)** Pathway module heatmap of scaled signature scores per cluster. Rows are individual signature *z*-score normalized and grouped by module, and columns are clusters. **(D)** Per-cluster proportion of cells originating from autoimmune (red) and viral (blue) samples, normalized to sample composition. FDR-adjusted *q*-values are shown at the right. **(E)** Within-cluster principal component projection (PC1 vs. PC2) of patient-level pseudobulk profiles for the seven clusters with sufficient cells (clusters 6 and 8 omitted for low representation). Cluster N includes: 0 (NIU = 12, viral = 12); 1 (4,12); 2 (4,12); 3 (2,12); 4 (5,8); 5 (8,8); 7 (5,3). Each point is one patient. Ellipses indicate 95% confidence regions for autoimmune (red) and viral (blue). Dashed lines mark zero. **(F)** PC1 loadings for genes in T-cell-instructive programs, restricted to the clusters with significant autoimmune-vs.-viral separation in **(E)**. Each point is a single gene’s PC1 contribution, faceted by program and colored by cluster. Negative loadings indicate genes that drive cells toward the autoimmune pole of PC1; positive loadings indicate genes that drive cells toward the viral pole.

Subset abundance differed sharply between conditions (**Figure 3D**). The DC compartment skewed toward autoimmune disease, with pDCs, Langerhans-like/cDC2, and cDC1 all significantly enriched in autoimmune samples (both at *q <*4e-4). Classical inflammatory monocytes and intermediate/transitional monocytes showed a preference for viral samples, consistent with acute myelopoietic recruitment during infection. *FOLR2*+ TRM macrophages and *TREM2*+ LAM showed milder, condition-specific shifts. Beyond differential abundance, disease etiology imposed a second, orthogonal layer of variation. Despite clustering together, autoimmune and viral cells occupied non-overlapping regions of the intracluster principal component space (**Figure 3E**; **Supplementary Figure S3**). The separation held across inflammatory monocytes, *FOLR2*+ TRM and *TREM2*+ LAM macrophages, the residual macrophage population, pDC, Langerhans/cDC2, and cDC1. Patient samples from the two conditions formed distinct 95% ellipses on PC1 and PC2, indicating that disease etiology reshapes the transcriptomes of each subset, even when the same cell type is present in both settings. These transcriptional shifts converged on programs that drive T-cell instruction (**Figure 3F**). Autoimmune myeloid cells showed a trend toward higher HLA-II and costimulatory module scores, particularly in DC subsets, a phenotype that would support effector T-cell licensing (**Figure 3F**). Viral myeloid cells showed higher HLA-I and selected inhibitory ligand/receptor scores in the monocyte and macrophage compartments, consistent with CD8 priming and counter-regulation during acute infection. Cytokine and chemokine programs are split by both cluster and disease, with autoimmune-biased signals in Langerhans/cDC2 and cDC1 and viral-biased signals in inflammatory monocytes (**Figure 3F**). These data together show that intraocular myeloid cells differ between autoimmune and viral uveitis at three levels: which subsets are present, the intrinsic transcriptional programs that each subset runs, and the T-cell-instructive surface signals that they display.

### T cells mirror the myeloid disease axis within patients

To further investigate the differential program between autoimmune and viral uveitis, we next focused on the sub-analysis for the intraocular T-cell compartment. This analysis recovered six clusters spanning the conventional CD4 and CD8 landscape (**Figure 4A**): naive/CM CD4 (cluster 0), cytotoxic CD8 EM/CTL (cluster 1), CD4 Tfh-like marked by *CXCL13* and *PDCD1* (cluster 2), effector memory T expressing *ITGA1* (cluster 3), Treg (cluster 4), and cycling T cells (cluster 5). Canonical markers separated the clusters cleanly (**Figure 4B**). *CD4* and *IL7R* marked the naive/CM and Tfh populations, *CD8A* with *GZMK* and *GZMB* marked the cytotoxic compartment, *CXCL13* and *PDCD1* identified the Tfh-like, *ITGA1* and *CXCR6* marked tissue-resident effector memory, *FOXP3* marked Tregs, and *MKI67* marked cycling cells.

**Figure 4:**
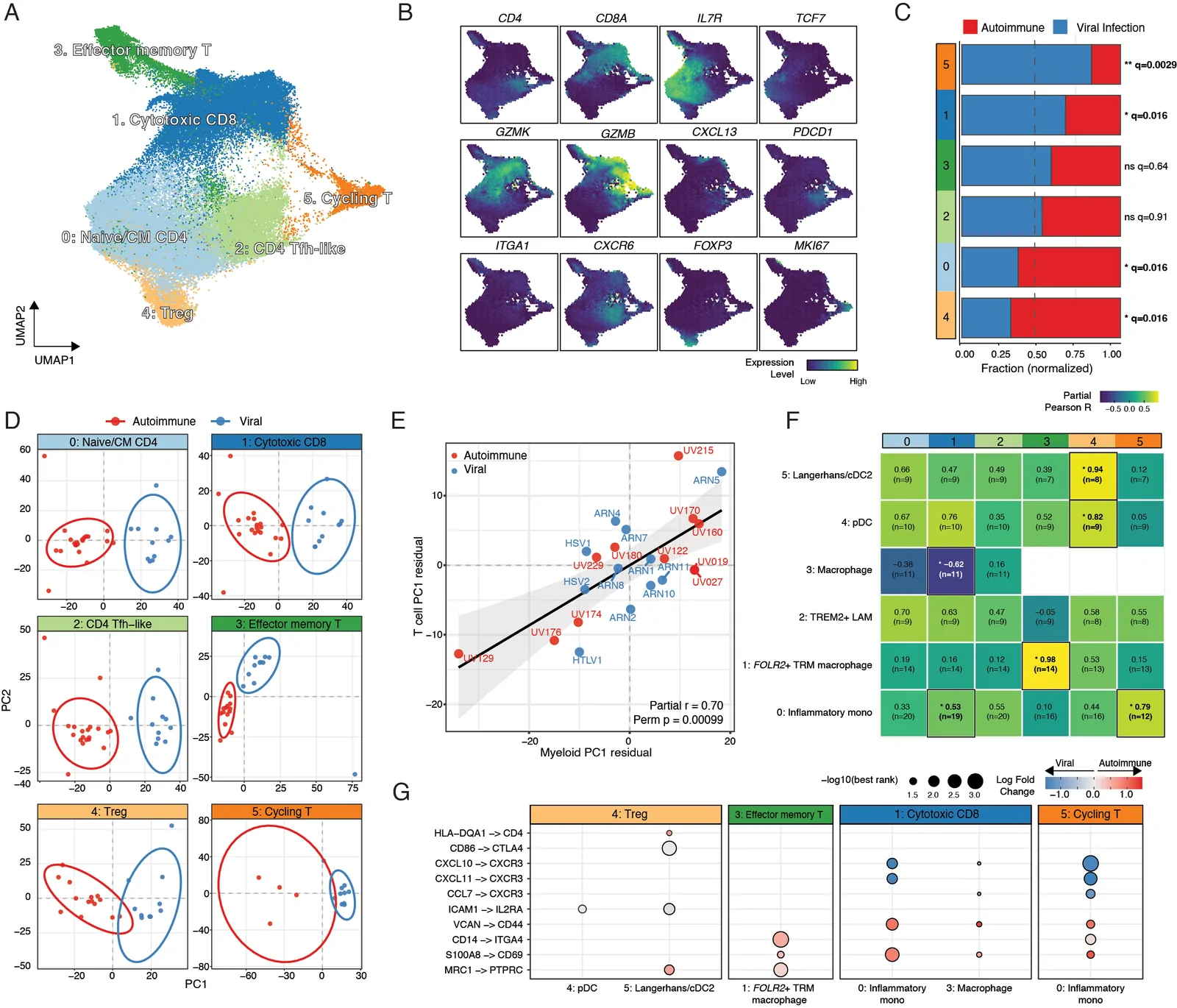
Intraocular T cells mirror the myeloid disease axis and couple to myeloid programs within patients. **(A)** UMAP embedding of intraocular T cells (*n* = 72,239 cells) aggregated across autoimmune and viral uveitis samples, colored by unsupervised cluster. **(B)** Feature plots of canonical T cell markers projected on the UMAP from **(A)**. Markers cover naive/CM (*CD4*, *CD8A*, *IL7R*, and *TCF7*), cytotoxic effector (*GZMK* and *GZMB*), Tfh/Tex (*CXCL13* and *PDCD1*), tissue residence (*ITGA1* and *CXCR6*), Treg (*FOXP3*), and cycling (*MKI67*). **(C)** Per-cluster proportion of cells originating from autoimmune (red) and viral (blue) samples, normalized to sample composition. FDR-adjusted *q*-values are shown at the right. **(D)** Within-cluster principal component projection (PC1 vs. PC2) of patient-level pseudobulk profiles per T cell cluster. Each point is one patient. Ellipses indicate 95% confidence regions for autoimmune (red) and viral (blue). **(E)** Per-patient myeloid PC1 residual versus T-cell PC1 residual. Each point is one patient labeled by sample ID, colored by etiology. The black line is OLS regression across all subjects with 95% confidence band [0.439, 0.841]. **(F)** Pairwise Pearson correlation matrix of subject-level PC1 scores across myeloid clusters (rows) and T-cell clusters (columns). Asterisks mark FDR-adjusted significance (*q* < 0.05). *n* per cell reflects subjects with sufficient cells in both clusters. Blank cells indicate fewer than the minimum number of paired subjects. **(G)** Bridge-conditioned ligand-receptor dot plot summarizing LIANA-predicted myeloid–T-cell signaling at the six significantly coupled cluster pairs from **(F)**. Dot size encodes –log10(best LIANA aggregate rank) across the autoimmune and viral conditions. Dot color encodes the per-pair logFC bias between conditions: autoimmune-driving interactions (red), viral-driving interactions (blue), and interactions of comparable strength in both conditions (greygray).

Disease etiology drove strong cluster-level shifts in abundance (**Figure 4C**). Cytotoxic CD8 and cycling T cells were enriched in viral samples, while Treg and Naïve/CM leaned toward the autoimmune conditions. Tfh-like and *ITGA1*+ effector memory compartments showed milder, condition-specific shifts. These compositional changes recapitulate the cell-type-level shifts in **Figure 2** at higher resolution. Pseudobulk PCA on subject-level expression profiles within each cluster separated autoimmune from viral subjects along PC1 in five of the six clusters (**Figure 4D**), with the cleanest separation in cytotoxic CD8 and CD4 Tfh-like. The same disease axis is, therefore, present in both compartments; however, whether it is coupled at the patient level was not clear.

To determine whether the T-cell and myeloid transcription programs were connected, we computed each subject’s myeloid PC1 residual and T-cell PC1 residual after removing the etiology mean from each compartment. By filtering the data to look only at variations within a single disease type, we eliminated the risk of the results being skewed by the natural differences that exist between various diseases. The myeloid PC1 residual and T-cell PC1 residual were strongly correlated across subjects (Pearson *r* = 0.70, *p* = 0.00099, *n* = 22; **Figure 4E**). Patients shifted toward the autoimmune pole in their myeloid compartment also shifted toward the autoimmune pole in their T-cell compartment, and the same held within each etiology arm. Because the pseudobulking approach with a small number of cells is potentially biased by outliers, we also evaluated rank-based correlation (Spearman *r* = 0.591, *p* = 0.0038, **Figure 4E**) and performed leave-one-subject-out partial correlations, finding minimal overall variability in the correlation trend and significance across all folds (**Supplementary Figure S4**). Decomposing the bridge to cluster-to-cluster resolution sharpened the picture (**Figure 4F**). The strongest couplings were *FOLR2*+ TRM macrophage with *ITGA1*+ effector memory T (*r* = 0.98, *q* < 0.05, *n* = 14), Langerhans/cDC2 with Treg (*r* = 0.94, *q* < 0.05, *n* = 8), pDC with Treg (*r* = 0.82, *q* < 0.05, *n* = 9), and inflammatory monocyte with cycling T (*r* = 0.79, *q* < 0.05, *n* = 12). The macrophage cluster showed a significant negative coupling with cytotoxic CD8 (*r* = −0.62, *q* < 0.05, *n* = 11). The pattern is structured rather than uniform, which argues against a generic technical confound and in favor of cluster-specific instruction. To identify the molecular wiring behind these cluster-level couplings, we ran LIANA (24) on myeloid-to-T-cell ligand–receptor interactions per disease arm and restricted the call set to the six significant cluster pairs from **Figure 4F** (**Figure 4G**, **Supplementary Table S6**). The analysis returned a structured vocabulary rather than a long tail of one-off interactions, with 30 of the top 40 ligand–receptor axes recurring across two or more coupled cluster pairs and six axes (*LYZ*➔ITGAL, *VCAN*➔ITGA4, *VCAN*➔ITGB1, *S100A8*➔CD69, *CXCL10*➔CXCR3, and *TNFSF13*➔TNFRSF14) appearing in at least four of the six pairs (**Supplementary Figure S5**). Against this shared background, autoimmune-biased instruction concentrated in dendritic-to-Treg signaling, where the Langerhans/cDC2➔Treg pair carried *CD86*➔CTLA4, *CD86*➔CD28, and *HLA-DQA1*➔CD4, and a broader *MHC-II*➔LAG3 checkpoint motif (*HLA-DPA1*, *HLA-DQA1*, and *HLA-DQB1*➔LAG3) recurred across three coupled pairs, matching the elevated HLA-II and costimulatory module scores in autoimmune DC subsets (**Figure 3F**). Viral-biased signaling concentrated in the inflammatory monocyte➔cytotoxic CD8 and inflammatory monocyte➔cycling T pairings, where the full CXCR3 chemokine repertoire (*CXCL9*, *CXCL10*, *CXCL11*, and *CCL7*) dominated. The disease signal, therefore, resides in the costimulatory and chemokine arms of the myeloid–T-cell conversation rather than in the shared contact infrastructure, motivating further investigation into the T-cell compartment to assess antigen specificity via the TCR.

### Convergent intraocular TCRs identify candidate autoimmune-pathogenic clones that engage the myeloid signaling axis

The myeloid–T-cell coupling defined in **Figure 4** motivated a deeper look at the T-cell receptor repertoire. Intraocular TCR repertoires differed between viral and autoimmune uveitis, with viral samples having a significantly higher Gini coefficient, consistent with greater clonal expansion from an antiviral response (**Supplementary Figure S6A**). There was a trend toward greater sharing of intraocular expanded clones with the blood compartment in the autoimmune (35%) versus viral (21%); however, this was not significant (**Supplementary Figure S6B**). Within the NIU etiologies, there were varying levels of clonal sharing across the blood and intraocular compartments, which appeared to correlate with the relative TCR repertoire expansion in the blood (**Supplementary Figures S6C, D**). We next asked whether intraocular T cells carrying viral antigen specificities populated the clusters we already understood. VDJdb annotation (25) recovered five high-coverage pathogen specificities (CMV, EBV, influenza A, SARS-CoV-2, and HTLV-1), with HTLV-1 clones exclusive to HTLV-1-associated uveitis as expected (**Figure 5A**). Investigations into VZV- and HSV1/2-associated TCRs were limited by the overall low number of annotated sequences for these etiologies in the VDJdb (**Supplementary Table S7**). The cluster composition of pathogen-annotated clones differed sharply between disease arms (**Figure 5B**). In viral samples, 74% of these clones occupied the cytotoxic CD8 cluster, the expected anti-viral effector phenotype (**Supplementary Figure S6E**). There was no trend for the enrichment of clones for specific viral sequences and the viral etiology of uveitis. In autoimmune samples, the same specificities distributed across the naive/CM CD4, Tfh-like, and effector memory compartments, consistent with bystander or memory-resident function rather than active effector function. To anchor the framework on a known autoreactive T cell, we tested whether the published HLA-B*27 pathogenic TCR motif (TRAV21 paired with a CDR3-beta [YF]S[TS] core) was detectable intraocularly (34). This TCR motif is enriched approximately 10–100-fold in the eyes of patients with HLA-B*27^+^ acute anterior uveitis (in the presence or absence of concurrent ankylosing spondylitis) as well as the joint of patients with ankylosing spondylitis, suggesting multi-organ self-reactivity (26). This pathogenic TCR motif was able to identify 54 ocular T cells, which were found in the Tfh-like CD4 T cell (*n* = 35) and cycling T clusters (*n* = 9, **Figures 5C, D**). The per-subject fraction of ocular T cells carrying the motif was significantly higher in HLA-B*27-positive uveitis than in either HLA-B*27-negative uveitis or viral uveitis (Wilcoxon *p* = 0.00137 and *p* = 0.00185, **Figure 5E**). Thus, the HLA-B*27-associated TCR motif shows robust disease specificity but transcriptional plasticity similar to that of some CD4 T-cell subsets.

**Figure 5:**
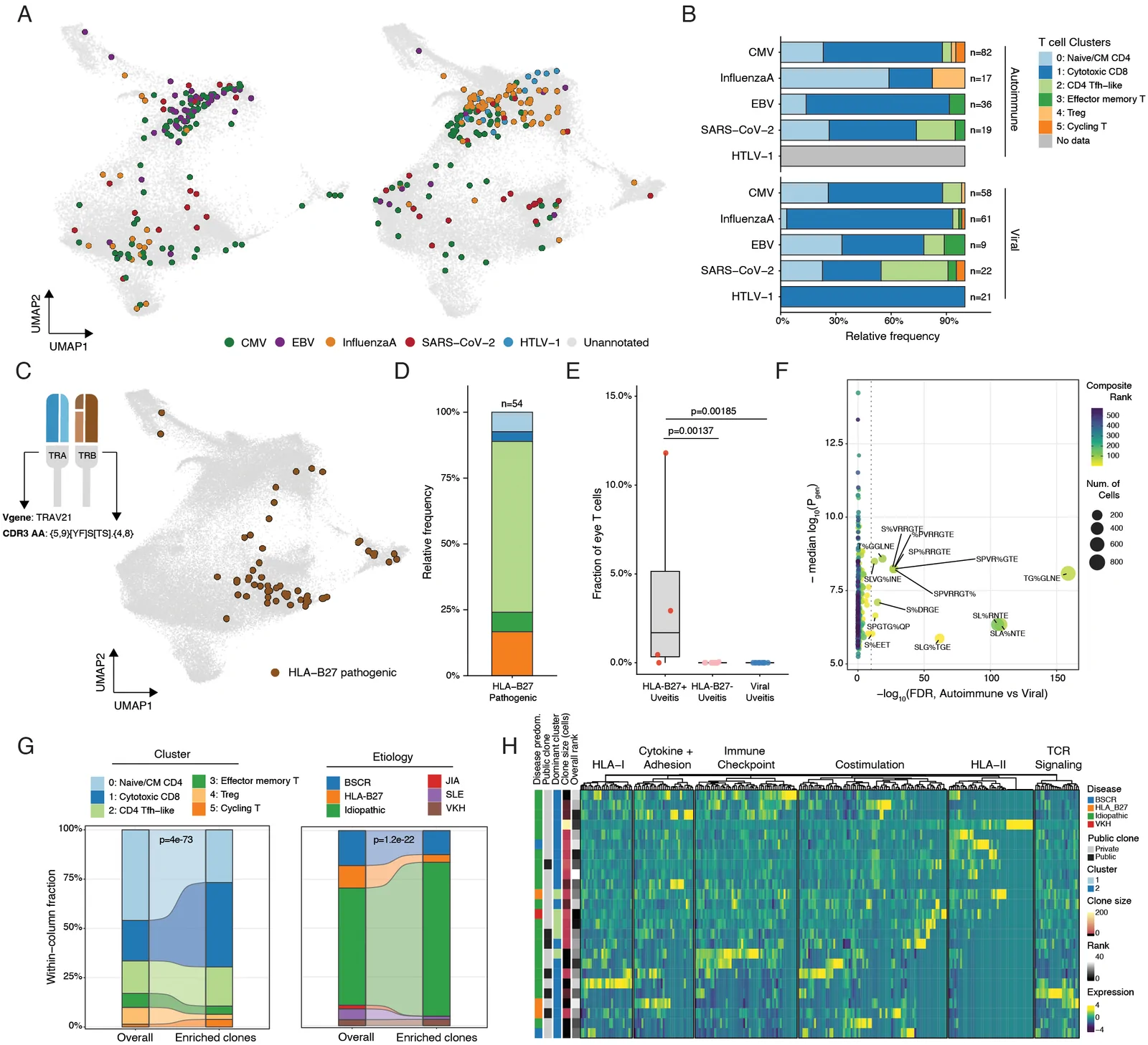
Antigen specificity and across intraocular TCRs and identification of autoimmune-pathogenic TCR motifs. **(A)** UMAP of intraocular T cells with VDJdb-annotated clones overlaid by pathogen species. Autoimmune subjects on the left and viral subjects on the right. **(B)** Cluster composition of pathogen-annotated clones by disease arm. Bars are within-pathogen relative frequency across the six T-cell clusters. **(C)** UMAP layout with clones carrying the published HLA-B*27 pathogenic TCR signature highlighted (20). **(D)** Cluster composition of the HLA-B*27 pathogenic clones (*n* = 54 cells). **(E)** Per-subject fraction of ocular T cells carrying the HLA-B*27 signature in HLA-B*27+ uveitis, HLA-B*27− uveitis, and viral uveitis. Individual points represent patients. Two-sided Wilcoxon *p*-values are shown. **(F)** Each point is a GLIPH motif cluster scored on a composite of 11 features spanning ranking with autoimmune-versus-viral cell counts (x-axis) and generation probability (y-axis). **(G)** Alluvial comparisons of where the top-100 clonal candidates’ cells sit across the cohort. Left stratum, all NIU ocular T cells; right stratum, cells belonging to the top-100 CDR3 candidates. Bar widths give within-column fractions. Overall difference in distribution between strata was tested with a Pearson *χ^2^* test of independence on the 2 × K candidate-vs.-non-candidate table. **(H)** Per-clone engagement of the autoimmune myeloid signaling vocabulary. Rows are individual CDR3-beta clones from the top 25 candidates, and clone size is based on the number of CDR3 sequences within the individual intraocular sample. Columns are genes drawn from LIANA pairs that are significantly autoimmune-biased (CellPhoneDB *p <*0.05 and disease_bias_logfc >0).

This positive control sets up a discovery question: are intraocular T cells carrying sequence-convergent motifs whose autoimmune association is novel? We scored every GLIPH-2 motif cluster on a composite of 11 features spanning generation probability, NIU-versus-viral Fisher discrimination, GLIPH convergence breadth across subjects, TCRdist network centrality, MiloR differential abundance of the dominant T-cell cluster, and the cell’s engagement of the myeloid–T-cell receptor module (see “Methods”). The HLA-B*27 motif was retained as a benchmark during sweep diagnostics and excluded from the discovery panels. Sweeping the top-N cutoff from 10 to 577 candidates confirmed that the directional findings were stable across cutoff choice (**Supplementary Figure S7**). The discovery landscape places motifs with strong NIU discrimination to the right and antigen-selected (low-Pgen) motifs upward (**Figure 5F**). Defining the location of these candidate cells revealed a divergence from the cluster-abundance findings of **Figure 4**. Cells carried by the top 100 clonal candidates were enriched in cytotoxic CD8 (OR = 3.0, FDR = 2e-41) and cycling T (OR = 3.0, FDR = 3e-6) and depleted in Treg (OR = 0.29, FDR = 3e-10) and naive/CM CD4 (OR = 0.42, FDR = 6e-26) (**Figure 5G**, left). Treg and naive/CM CD4 were the autoimmune-leaning clusters at the cluster-abundance level, yet the convergent sequence-defined candidates do not reside there. By indication, candidate cells were enriched in idiopathic uveitis (OR = 2.5, FDR = 3e-26) and depleted in HLA-B*27, systemic lupus erythematosus (SLE), juvenile idiopathic arthritis (JIA), and birdshot chorioretinitis (BSCR) (**Figure 5G**, right). The discovery framework, therefore, prioritizes cross-patient convergent clonotypes shared by idiopathic uveitis rather than the indication-specific clones associated with named autoimmune diagnoses. These data suggest that sub-groups of idiopathic uveitis may share antigenic triggers.

To couple the candidate clones back to the myeloid signaling vocabulary (**Figure 4F**), we computed each top TCR candidate’s per-cell expression of every gene drawn from the LIANA pairs that were significantly autoimmune-biased (**Figure 3E**) (see “Methods”). After removing zero-variance genes, the remaining informative profiles were *z*-scored within columns and clustered by Ward-D2 linkage (**Figure 5H**). Hierarchical column splitting recovered six functional modules whose dominant LIANA ligand-family annotations identified them as HLA class I antigen presentation, cytokine and adhesion signaling, immune checkpoint, co-stimulation, HLA class II antigen presentation, and TCR signaling. Top candidate clones engaged the immune checkpoint, co-stimulation, and HLA class II modules at the highest *z*-score, recapitulating the exact arms that distinguished autoimmune dendritic-cell signaling in **Figure 4G**. The HLA class I and cytokine/adhesion modules showed sparse, clone-specific engagement, while the TCR signaling module was uniformly elevated as expected for any T cell.

We next ranked TCRs based on disease association (NIU or viral), how often TCRs were found within and across individuals, how often the TCRs were enriched in the eye over the blood, upregulation of genes associated with NIU or viral uveitis, and how connected the cells were within the larger network of T-cell patterns. Two clones emerged as the highest-confidence individual candidates by combined composite rank and module engagement (**Supplementary Table S3**). Two convergent GLIPH motifs scored highest for combined autoimmune discrimination and receptor-module engagement: S%EET_KQRS (four distinct rearrangements across six subjects) and SLG%TGE_AGTV (four rearrangements across four subjects). Both motifs were over 90% NIU-derived and dominated by cytotoxic CD8 T cells (cluster 1). Engagement and discrimination are reported at the motif level because the per-clone values were computed on single expanded clones from individual subjects. These data identify shared TCR motifs across individuals with non-infectious uveitis, suggesting common uveitic antigens in the anterior uveal tissues.

### B-cell compartments diverge in BCR lineage architecture but not transcriptional state

To ask whether the disease axis observed in the myeloid and T-cell compartments extended to humoral immunity, we focused on sub-clustering within the B and plasma cell lineages. Five populations resolved across the integrated eye and blood data: plasma cells (cluster 0), *MS4A1*^+^ *ITGAX*^+^ memory B cells (cluster 1), cycling plasmablasts (cluster 2), IgG plasma cells (cluster 3), and cycling memory B cells (cluster 4) (**Figure 6A**). Cluster-defining markers separated the populations, with *JCHAIN*, *DERL3*, *FKBP11*, *IGLL5*, and *MZB1* anchoring the plasma and plasmablast states and *MS4A1*, *AFF3*, *CD24*, *GAPT*, and *ITGAX* anchoring the memory compartment. The two cycling clusters carried a coherent cell-cycle module spanning *RRM2*, *CCNA2*, *TOP2A*, *CEP55*, *ASPM*, and *CENPF* (**Figure 6B**). Compositional testing revealed a disease-stratified skew across three of the five populations. Memory B (*q* = 0.013), cycling plasmablast (*q* = 0.0016), and IgG plasma cells (*q* = 0.0014) were significantly biased between viral and autoimmune samples, while bulk plasma cells and cycling memory B cells were not (**Figure 6C**).

**Figure 6:**
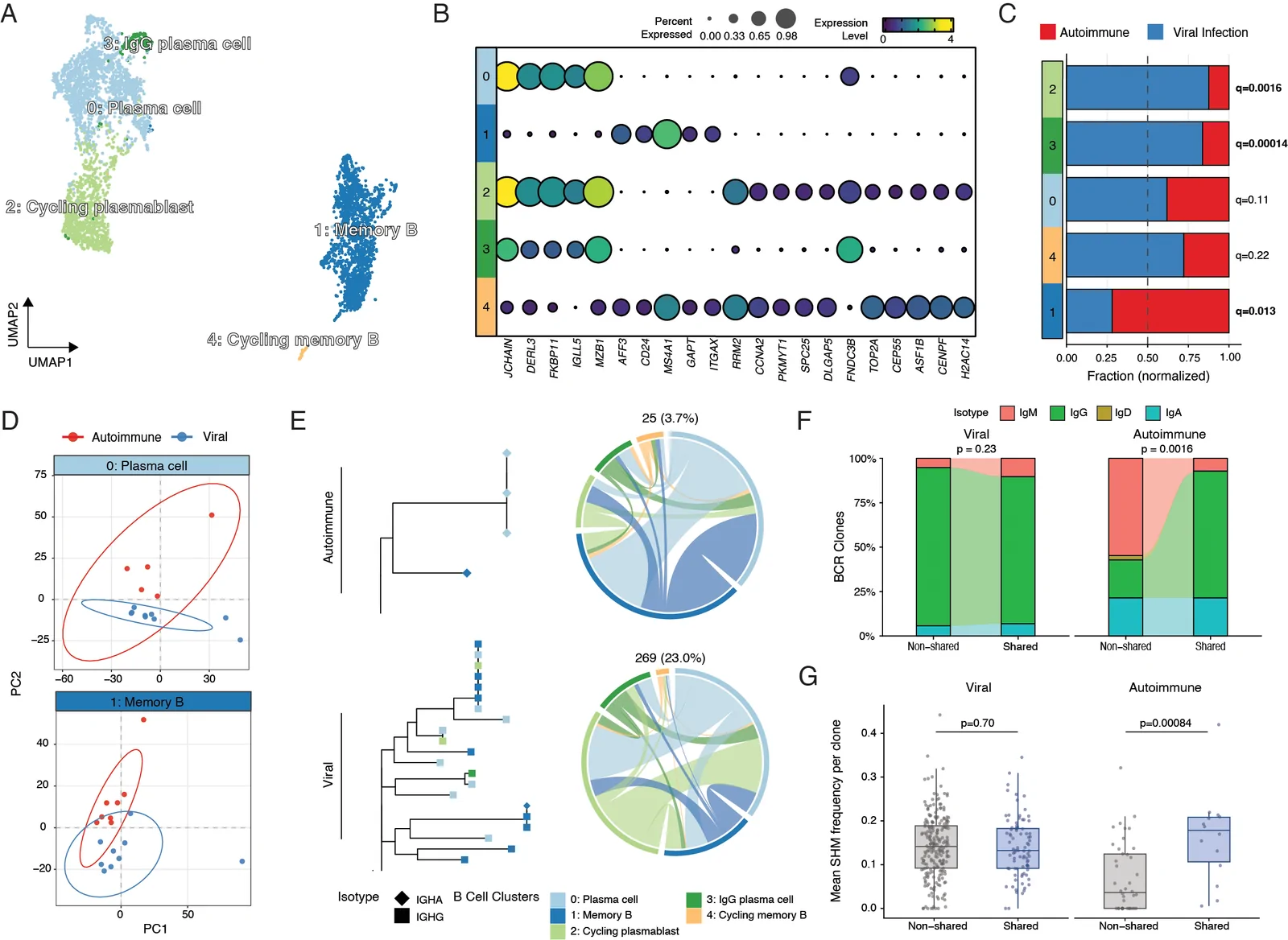
B-cell compartments share transcriptional state across disease contexts but diverge in BCR lineage architecture. **(A)** UMAP of paired eye and blood B cells, colored by cluster. **(B)** Dot plot of cluster-defining markers. Dot size encodes the fraction of cells expressing each gene, and color encodes scaled mean expression. **(C)** Normalized fraction of cells per cluster contributed by autoimmune (red) and viral infection (blue) donors. The adjusted *q*-values are from a per-cluster proportion test against the global disease ratio (dashed line). **(D)** Principal component analysis of pseudobulk transcriptional profiles for plasma cells (cluster 0, NIU = 5, and viral = 9) and memory B cells (cluster 1, NIU = 8, and viral = 9), with 95% confidence ellipses by disease. **(E)** Architecture of expanded BCR lineages. Left, representative maximum-parsimony lineage trees from an autoimmune donor (top) and a viral donor (bottom), with tips shaped by isotype (IgA, diamond; IgG, square) and colored by B-cell cluster. Right, chord diagrams summarizing cluster-to-cluster sharing of clonal members across all expanded lineages within each disease group. **(F)** Within-column composition of clone-dominant isotype across non-shared and shared clones, faceted by disease. Shared clones span at least two B-cell clusters. Alluvial ribbons connect matched isotype proportions between the two columns. Overall difference in distribution between strata was tested with a Pearson *χ^2^* test of independence. **(G)** Per-clone mean somatic hypermutation frequency, split by non-shared and shared clones, faceted by disease.

In contrast to the myeloid and T-cell compartments, this compositional shift did not track with a coherent transcriptional program. Pseudobulk principal component analysis of the two largest populations, plasma cells and memory B cells, placed autoimmune and viral samples in overlapping regions along the leading components, with no clean disease-specific separation in either compartment (**Figure 6D**). The disease signal instead emerged at the level of BCR lineage architecture. Maximum-parsimony reconstruction of expanded clones showed that the autoimmune lineages were small and largely confined to memory B or plasma cell clusters, while viral lineages were larger and spanned memory B and plasma cell compartments. Quantifying cross-cluster sharing across all expanded lineages confirmed that viral BCRs were strongly enriched for cells participating in multi-cluster trees relative to autoimmune (269 vs. 25 cells, 23.0% vs. 3.7%). After controlling for clone size, viral phenotype carried a size-adjusted OR of 2.98 (*β* = 1.09, *p* = 1.3e-4) by logistic regression (**Figure 6E**). At any given clone size, a viral clone was roughly three times more likely to span multiple B-cell clusters than an autoimmune clone, indicating that the architectural difference is not explained by viral lineages simply being larger.

To ask whether the architectural difference reflected biased recruitment from particular clonal subsets, we compared dominant isotype and somatic hypermutation between shared (multi-cluster) and non-shared clones within each disease. In viral samples, shared and non-shared clones carried nearly identical isotype distributions, with class-switched IgG dominating both groups (*χ*^2^ = 2.87, *p* = 0.23). Shared autoimmune lineages were strongly enriched for IgG, while non-shared autoimmune clones retained substantial IgM and IgA representation (*χ*^2^ = 13.6, *p* = 0.0016) (**Figure 6F**). The same pattern appeared in somatic hypermutation (SHM). Viral clones carried comparable mean SHM whether or not they spanned multiple clusters (*W* = 10,197, *p* = 0.70), whereas in autoimmune samples, shared clones carried substantially higher SHM than non-shared clones (*W* = 119, *p* = 8.4e-4, Cliff’s *δ* = −0.60, large effect) (**Figure 6G**). At the cohort level, viral subjects trended toward higher per-subject mean SHM than autoimmune subjects, though this contrast did not reach significance at *n* = 13 (*W* = 7, *p* = 0.067, Cliff’s *δ* = −0.65). Autoimmune cross-cluster trafficking was, therefore, restricted to mature, class-switched, affinity-matured lineages. In viral uveitis, the broader clonal repertoire, including less mature clones, participates evenly in cross-cluster expansion. Taken together, these data suggest that rather than mounting transcriptionally distinct effector programs as the myeloid and T-cell compartments do, B-cell responses diverge in how clonal families are organized across functional states, with viral infection driving broader cross-state lineage trafficking and autoimmune disease favoring restricted, plasma-cell-centered expansion.

## Discussion

Herein we provide the first single-cell atlas comparing ocular immune cells from non-infectious and viral uveitis. Viral uveitis was characterized by an influx of monocytes, macrophages, and cytotoxic CD8 T cells. Our data further suggest that, in viral uveitis, these myeloid cells may be the major source of T-cell activation through antigen presentation via MHC-I while concurrently providing inhibitory receptor signaling to limit inflammation. In contrast, our data suggest that non-infectious uveitis is characterized by an influx of dendritic cells that appear to activate both effector T_H_17 CD4 T cells and Treg responses via antigen presentation by MHC-II. In parallel, pairing VDJ sequencing with gene expression revealed that idiopathic non-infectious uveitis may have a greater enrichment of T-cell clones than other forms of non-infectious uveitis, while B-cell clonotypes in viral uveitis undergo greater clonal expansion and somatic hypermutation. Collectively, these data highlight the divergent potential mechanisms of ocular injury from infectious and non-infectious etiologies.

Prior scRNAseq analysis of ocular immune cells have focused on non-infectious uveitis and the ocular composition or enrichment of immune cells in the eye (1–5). However, our comparison of immune responses between infectious and non-infectious etiologies offers an important approach to define the key features of each process. As expected, viral uveitis was characterized by a signature of interferon signaling, with a particular connection between the myeloid cell expression of CXCR3 ligands (*CXCl10*, *CXCL11*, and *CCL7*) and *CXCR3* expression on cytotoxic CD8 T cells and cycling T cells. In contrast, non-infectious uveitis demonstrates greater networking between T cell and myeloid cells via adhesion pathways, including *CD44*, *CD45*, and *ITGA4*. These differences likely reflect the uniformity of type 1 antiviral responses versus the variety of autoimmune responses that may include a mixture of type 1 and type 17 immune cells.

Our data suggest that a major mechanism in non-infectious uveitis is a type 17 response, which is regulated by RORC. The failure of an IL-17A inhibitor, secukinumab (35), to ameliorate non-infectious uveitis across three separate clinical trials has been unexpected. This discrepancy is seen in other human inflammatory conditions, such as inflammatory bowel disease, where type 17 T cells appear to promote disease in human studies and animal models, but the inhibition of IL-17A failed to improve disease activity (36, 37). However, prior studies have reported that type 17 T cells can drive autoimmunity even after IL-17 deletion, such as T_H_17 cells in animal models and human disease (38–40). Therefore, pathogenic type 17 T cells can mediate autoimmunity through IL-17A-independent mechanisms.

While T_H_17 CD4 T cells are named for their production of IL-17, T_H_17 cells produce additional proinflammatory cytokines, such as GM-CSF (40). GM-CSF is pathogenic in several animal models (41). Moreover, inhibition of GM-CSF has demonstrated efficacy in human inflammatory disease, such as rheumatoid arthritis (42, 43). A challenge in single-cell analysis, however, is the poor sensitivity of cytokine detection in pathogenic populations for determining candidate cytokines for inhibition.

In addition to the production of multiple type 17 cytokines, T_H_17 cells can undergo additional differentiation and acquire aspects of type 1 immunity. These pathogenic T cells (referred to as T_H_1/17 or T_H_1* cells) co-produce IL-17A and IFNg as well as other pro-inflammatory cytokines such as TNFa (44, 45). These cells have been identified in animal models of EAE as well as human disease and appear to differentiate under the influence of IL-23 signaling (46). Thus, JAK inhibitors, which block a multitude of cytokine signaling pathways, including interferons and IL-23, may be better positioned to treat non-infectious uveitis than IL-17A inhibitors. The efficacy of JAK inhibitors for non-infectious uveitis was first tested in the phase II HUMBOLT trial (47). Although the study was terminated early for business reasons and only enrolled 74 participants out of the 110 initially planned, filgotinib reduced uveitis flare rates from 67% to 38% when compared to placebo. This rate of treatment response parallels the TNF inhibitor adalimumab, which reduced the rate of uveitis flares by half in the VISUAL-1 trial (48). Although filgotinib is no longer in development for the treatment of non-infectious uveitis, a second JAK inhibitor, brepocitinib, is currently being tested in a larger phase III trial of 371 participants (NCT06431373), which may offer clearer evidence regarding the efficacy of JAK inhibitors in the treatment of non-infectious uveitis.

In our TCR analysis, we found shared TCR motifs across samples from idiopathic uveitis. This finding is consistent with the model that a common antigenic stimulation may contribute to specific sub-groups of idiopathic uveitis and parallels prior work in HLA-B*27-associated acute anterior uveitis (26, 34). Larger cohorts of TCR sequencing of idiopathic uveitis samples, either through single-cell TCR sequencing of ocular fluids or high-throughput TCR sequencing of blood specimens, will likely allow for greater refinement of pathogenic TCR motifs and identification of distinct uveitic etiologies.

In this study, we used TCR motifs from HLA-B*27-associated acute anterior uveitis as a benchmark for autoreactive T cells. These disease-associated TCRs are known to come from CD8 T cells that respond to HLA-B*27-restricted bacterial and self-peptides (26, 34). However, in our dimensionality reduction, this TCR motif was spread across clusters containing cytotoxic CD8 T cells, T_FH_-like CD4 T cells, and cycling T cells. Although the specific function of the CD8 T cells carrying these disease-associated TCRs has yet to be defined, our data suggest that these autoreactive CD8 T cells do not acquire the traditional cytotoxic programming seen in anti-viral immunity but rather may develop into a type 17 CD8 T cell that has more similar transcriptional programming to T_FH_ or T_H_17 CD4 T cells.

Part of our TCR analysis utilized mapping to known TCR specificities through VDJdb. TCRs submitted to VDJdb are weighted toward CD8 T cells and anti-viral TCRs, including specificities for VZV, CMV, HTLV-1, HSV-1, HSV-2, EBV, influenza A, and SARS-CoV-2. For these antigens, the CD8 to CD4 bias was approximately 20:1 in terms of the known HLA-specific TCRs. Thus, our TCR mapping may have had more hits for the anti-viral TCRs and allowed for a deeper analysis, given the limitations of known TCR specificities.

Our computational comparisons have several clinical confounders that we cannot resolve in this current dataset. Specifically, the samples from non-infectious uveitis were collected from the anterior chamber at a single center within the United Sates, whereas the samples from viral uveitis were collected from the vitreous in Japan. One area where this may have had an impact is on our analysis of T-cell and B-cell clonality, as the aqueous humor in the anterior chamber has more rapid turnover, while the collagen fibers and hyaluronic acid of the vitreous and the blood-retinal barrier lead to slower turnover (49). The slower turnover of the vitreous could lead to a greater concentration of clonally expanded lymphocytes by reducing the number of bystander lymphocytes transiting through the inflamed tissue. A second area of potential impact is in the elevation of stress response genes based on the differences of sample processing, e.g., vitreous samples were subjected to mechanical shearing and filtration. However, our integration excluded heat-shock, ribosomal, and mitochondrial genes from variable feature selection, meaning that stress response genes did not enter the embeddings underlying cell clustering. Moreover, of the 5,919 significant differentially expressed genes in ocular cells, only 74 (1.3%) were members of the van den Brink dissociation signature (50), suggesting that differences in processing had only a small impact on gene expression.

While an ideal study design would compare both anterior chamber and vitreous sampling in participants with either non-infectious uveitis or viral uveitis to control for the distinct anatomic environments, we were limited by ethical and practical considerations—for example, it is not ethical to perform a vitrectomy in patients with uncomplicated anterior uveitis. Nevertheless, we posit that the immunologic differences from autoimmune and viral etiologies far exceed potential differences between anatomic chambers of the eye, genetic background, or environmental risk factors. Future analysis of immune cells from the anterior chamber during viral infection may help to resolve this question.

An additional limitation of our analysis is in the pooling of multiple forms of non-infectious uveitis into a single NIU category for comparison with viral uveitis—for example, the immunologic mechanisms behind JIA uveitis, VKH, BSCR, and HLA-B*27-associated acute anterior uveitis are likely diverse and divergent from idiopathic disease. Specifically, the MHC class I associations for BSCR (HLA-A*29) and HLA-B*27-associated acute anterior uveitis may lead to a different cellular signature than VKH, which has an MHC class II association (HLA-DR4) (51). However, removing each NIU etiology and repeating the eye pseudobulk comparison did not meaningfully impact the list of differentially expressed genes (**Supplementary Figure S2**). Thus, differences between non-infectious and viral uveitis appear robust enough to overcome the noise generated by mixing several autoimmune etiologies.

Our TCR analysis was performed without complete knowledge of the HLA alleles of many participants, including the participants with viral uveitis. We found that “public” TCRs were not only shared across individuals for both HLA-associated diseases (such as HLA-B*27+ acute anterior uveitis) but also shared across individuals with idiopathic disease. Although HLA haplotyping could reinforce the conclusion of a shared peptide antigen across patients with uveitis, a single TCR can accommodate chemically similar amino acid side chains or homologous peptide sequences presented by separate, often structurally related, MHC allomorphs. Moreover, different HLA alleles within the same superfamily (such as HLA-A*02 subtypes or HLA-B alleles with overlapping anchor preferences) can present structurally comparable epitopes that fit the same TCR binding footprint. Thus, it is difficult to disprove that TCRs recognize disparate antigens in HLA-mismatched individuals.

This comparison between infectious uveitis and non-infectious uveitis revealed clear differences in the ocular immune response to self and foreign antigens. In our dataset, the cases of non-infectious etiologies were quite heterogeneous, spanning from systemic disorders, such as juvenile idiopathic arthritis, systemic lupus erythematosus, and Vogt–Koyanagi–Harada disease, to ocular restrictive disease, such as birdshot chorioretinitis or idiopathic uveitis. Infectious uveitis also has a diverse array of etiologies, spanning diverse viruses and bacteria. Thus, whether the ocular immune response to bacterial infections, such as *Borrelia burgdorferi* or *Mycobacterium tuberculosis*, resembles either non-infectious or viral uveitis or elicits a distinct immune response remains to be determined.

## Data Availability

FASTQ files for GEX are available through dbGaP at https://www.ncbi.nlm.nih.gov/projects/gap/cgi-bin/study.cgi?study_id=phs004518.v1.p1. Aligned and processed data is available at https://doi.org/10.5281/zenodo.20783773. All code for the analysis is publicly available at: https://github.com/BorchLab/Uveitis-Atlas.

## Supplementary material

Supplementary Figure 1 Hexbin-based UMAP plots show normalized expression of T cell (*CD3D*, *CD8A*, *CD4*, *FOXP3*), B and plasma cell (*MS4A1*, *MZB1*), myeloid (*CD68*, *FCGR1A*), cytotoxic and NK (*NKG7*, *KLRD1*), and plasmacytoid dendritic cell (*LILRA4*, *IL3RA*) genes, confirming spatial segregation of the major immune compartments.

Supplementary Figure 2 Leave-one-diagnosis-out sensitivity analysis of the intraocular autoimmune versus viral pseudobulk comparison (DESeq2, patient-level pseudobulks) for the overlap of differentially expressed genes (left), the log2FC correlation across folds (middle), and the overlap of the top 50 genes (right).

Supplementary Figure 3 Within-cluster principal component projection (PC1 vs. PC2) of patient-level pseudobulk profiles for all clusters without minimum cell floor. Each point is one patient. Ellipses indicate 95% confidence regions for autoimmune (red) and viral (blue).

Supplementary Figure 4 Leave-one-patient-out jackknife of the partial correlation between patient-level myeloid PC1 and T-cell PC1, controlling for disease group. Each point is the partial Pearson correlation, recomputed with that patient removed, across all 22 patients with paired myeloid and T-cell pseudobulks.

Supplementary Figure 5 Ligand-receptor interactions between myeloid and dendritic cell senders (bottom labels) and T-cell receivers (top labels), grouped by functional class. Dot color encodes the log fold change in interaction strength and dot size encodes significance as -log10 of the best rank.

Supplementary Figure 6 **(A)** Clonal expansion of TCR repertoires shown as Lorenz curves plotting the cumulative fraction of cells against the top fraction of unique CDR3 sequences ranked by abundance. Thin lines show individual samples and thick lines show the group means. **(B)** Blood-to-eye sharing of expanded TCR clones. Each expanded eye clone (≥3 cells in the eye) is classified as shared (detected in the paired blood, indigo) or eye-restricted (not detected in blood, green). **(C)** Raw proportion (left) and Morisita index (right) of clonal sharing across the intraocular and peripheral blood repertoires by NIU indication **(D)** Gini coefficient of the TCR repertoires in the intraocular (left) and peripheral blood (right) by the NIU indication. **(E)** CD4/CD8 lineage of antigen-specific intraocular T cells. T cells annotated to a viral specificity by VDJdb matching are shown as the fraction assigned to CD8 (red), CD4 (blue), or cycling (orange) clusters, split by disease group (autoimmune vs. viral).

Supplementary Figure 7 Sensitivity of TCR motif enrichment to the number of candidate motifs retained. log2 odds ratios are plotted against the top-N motif cutoff, stratified by transcriptional cluster (top) and clinical indication (bottom), with filled points marking FDR < 0.05 and open points non-significant results.
